# Educational gradients and sex-by-education differences in environmental health literacy among adults from selected urban communities in Nanning, China

**DOI:** 10.3389/fpubh.2026.1928358

**Published:** 2026-10-02

**Authors:** Xia Luo, Huiting Nong, Yang Yu, Linhan Liang, Dan Gu, Li Chen, Huanhuan Chen, Zhi Li, Kang Li

**Affiliations:** 1 Department of Microbiology, School of Basic Medicine, Guangxi Medical University, Nanning, Guangxi, China; 2 Department of Respiratory and Critical Care Medicine, West China Hospital, Sichuan University, Chengdu, Sichuan, China; 3 Institute of Environmental Health and Endemic Disease Control, Guangxi Zhuang Autonomous Region Center for Disease Control and Prevention (Guangxi Zhuang Autonomous Region Academy of Preventive Medicine), Nanning, China; 4 Guangxi Key Laboratory of AIDS Prevention and Treatment, School of Public Health, Guangxi Medical University, Nanning, China; 5 Key Laboratory of Basic Research on Regional Diseases (Guangxi Medical University), Education Department of Guangxi Zhuang Autonomous Region, Nanning, China

**Keywords:** cross-sectional study, educational attainment, environmental health literacy, indirect association, sex-by-education interaction

## Abstract

**Background:**

Environmental health literacy (EHL) is essential for recognizing environmental risks and adopting health-protective behaviors. This study assessed EHL among adults recruited from selected urban communities in Nanning, Guangxi, China, examined sociodemographic correlates with emphasis on educational attainment and sex, and explored whether basic concepts were statistically linked to the association from environmental health knowledge to practical skills.

**Methods:**

A cross-sectional survey using non-probability, site-based convenience recruitment was conducted from November to December 2024 among eligible adults present at two selected urban community survey sites in Nanning, Guangxi, China. EHL was measured using a standardized 30-item instrument covering basic concepts, basic knowledge, and basic skills. Multivariable linear and logistic regression models were used to identify correlates of EHL and to test sex-by-education interactions. An exploratory bootstrap indirect-association analysis with 5,000 resamples assessed whether the association between basic knowledge and basic skills was statistically consistent with an indirect link through basic concepts.

**Results:**

Among 1,773 participants, 953 (53.75%) were female, and the mean total EHL score was 83.34 ± 10.91. In the analytic community sample, 1,574 participants (88.8%) met the 70-point criterion for sufficient EHL; the total-score distribution was left-skewed and concentrated above this threshold. Internal consistency was acceptable for the total instrument (Cronbach’s alpha = 0.718), whereas domain-specific coefficients were interpreted descriptively. Educational attainment showed the strongest and most consistent association with EHL. Compared with participants with primary school education or below, those with a master’s degree or above had an 11.32-point higher total score (95% CI: 7.84 to 14.80) and higher odds of sufficient EHL (aOR = 10.06; 95% CI: 3.82 to 28.98). Significant sex-by-education interactions were observed for total EHL, basic knowledge, and basic skills. The exploratory indirect association from basic knowledge to basic skills through basic concepts was small but statistically evident in the total sample and among women.

**Conclusion:**

Educational attainment was the strongest correlate of EHL in this selected urban community sample. The findings should be interpreted as sample-specific associations rather than population-representative estimates for all adults in Nanning. The observed sex-by-education heterogeneity and exploratory knowledge-concept-skill association suggest that EHL promotion should prioritize residents with limited education and use concept-centered, skill-oriented communication strategies.

## Introduction

Environmental degradation has emerged as a critical determinant of population health, with a substantial share of the global disease burden attributable to ecological instability and preventable environmental risks (1–4). The World Health Organization estimates that modifiable environmental factors, including chemical exposures, unsafe water, and air pollution, account for nearly one quarter of global deaths (5). Strengthening public understanding of how environmental conditions shape health is therefore not only an educational priority but also a core component of preventive public health.

EHL, brought to wider attention by the American Public Health Association in 2008, provides a conceptual bridge between environmental health science and health-protective action (6). It refers to the capacity to access, understand, appraise, and use information about environmental risks to protect individual and community health (6). By integrating principles from health literacy and environmental literacy, EHL encompasses both cognitive competencies and practical capacities, enabling individuals to recognize hazards, interpret risk information, and adopt behaviors that are beneficial to human health and ecological sustainability (7, 8).

Empirical research on EHL remains limited, particularly in China (9–11). Existing studies have primarily described EHL levels and their associations with socioeconomic status, household or indoor environmental exposures, and environmental risk awareness (7, 10–13). The 2018 national survey across 15 Chinese provinces reported that only 12.5% of residents achieved sufficient EHL (14). In response, the Healthy China Action (2019–2030) set a national target of increasing EHL to 25% by 2030 (15). This task is especially relevant in Guangxi, a region that functions as an important ecological barrier while facing environmental challenges related to karst groundwater vulnerability, industrialization, and transboundary water management (16–19). Improving EHL in this region is therefore important for both ecological security and health equity.

Although previous studies have increasingly characterized population differences in EHL, fewer have examined whether EHL subdomains are statistically linked to one another within a unified content framework (6, 10, 11, 20). Conceptually, EHL involves not only factual knowledge of environmental risks, but also risk appraisal, responsibility-related concepts, and the ability to apply information in protective action. Within the Chinese EHL assessment framework, basic knowledge, basic concepts, and basic skills are predefined content domains (21, 22). Examining the statistical interrelations among these domains may therefore help clarify how knowledge-related differences are associated with practical EHL skills. In addition, the joint influence of sex and educational attainment has rarely been examined within an interaction framework. To address these gaps, this study assessed EHL among adults recruited from two selected urban communities in Nanning, Guangxi, investigated whether education-related differences in EHL varied by sex, and explored whether basic concepts were statistically linked to the association between basic knowledge and basic skills. All analyses were interpreted as cross-sectional associations rather than causal pathways.

## Methods

### Study design and sampling

This study used a non-probability, site-based convenience recruitment strategy based on participant availability at designated community survey sites. The survey was conducted in Fenglingnan Community and Xiulingbei Community, two selected urban communities in Nanning, Guangxi, China, from November 15 to December 31, 2024. The two communities were purposively selected based on fieldwork feasibility, community willingness to participate, and the availability of local survey support. No household sampling frame was established, households were not sampled as a separate stage, and no systematic interval-based selection procedure was applied. The study was reported with reference to the STROBE recommendations for observational studies (23).

Eligible participants were permanent residents aged 18 years or older who had lived in the study area for at least 6 months and were able to complete a structured face-to-face questionnaire. During the survey period, trained investigators invited eligible adult residents who were present at the designated community survey sites to participate. Written informed consent was obtained before data collection.

A total of 1,773 participants with valid questionnaires were included in the final analysis, including 828 from Fenglingnan Community and 945 from Xiulingbei Community. Because participants were recruited from two selected community survey sites without probability sampling or population weighting, the findings should be interpreted as estimates for the analytic community sample rather than as representative estimates for all adults in Nanning.

### Questionnaire survey

The questionnaire consisted of two components: sociodemographic information and an EHL assessment. The EHL assessment was based on the national technical framework for citizen environment and health literacy assessment and the official statement on Ecological Environment and Health Literacy of Chinese Citizens issued by the Ministry of Ecology and Environment of the People’s Republic of China (21, 22). The instrument covers three predefined content domains. The Basic Concepts domain assesses cognition, attitudes, and perceived responsibilities concerning the relationship between the environment and health. The Basic Knowledge domain captures scientific and behavioral knowledge related to environmental risks, including noise, radiation, climate change, and air pollution. The Basic Skills domain evaluates the ability to obtain, interpret, and apply environmental health information in protective behaviors. These domains were treated as predefined content domains for scoring and analysis rather than as newly derived latent constructs. Internal consistency was assessed using Cronbach’s alpha for the total instrument and each domain.

### Scoring criteria

The instrument contained 30 scored items, including 20 single-choice and 10 multiple-choice questions, with a maximum total score of 100. Domain-specific scores for basic concepts, basic knowledge, and basic skills were calculated according to the scoring scheme of the survey instrument. A total score of 70 or above was used to define sufficient EHL according to the national assessment criterion (21).

### Statistical analysis

Analyses were performed using R software, version 4.5.1 (24). Categorical variables were summarized as frequencies and percentages, and continuous variables as means and standard deviations. Missing values were assessed before analysis; participants with missing values in variables required for a specific model were excluded from that model. The distribution of continuous EHL scores was examined using descriptive distributional statistics and histogram-based visualization. Because the total EHL score was a standardized summary score and the sample size was large, means and standard deviations were retained for descriptive reporting. The total-score distribution was left-skewed and concentrated above the 70-point threshold; this distributional pattern is shown in Supplementary Figure S1. Between-group differences in mean EHL scores were assessed using independent-samples t tests or one-way analysis of variance, as appropriate. Differences in the proportion of participants with sufficient EHL were examined using chi-square tests. Multivariable linear regression models were used for total and domain-specific EHL scores, which were treated as continuous outcomes. Multivariable logistic regression was used for sufficient EHL as a binary outcome. Models adjusted for age, sex, marital status, educational attainment, occupational exposure, and occupational safety and health protection. To evaluate effect modification, sex-by-education interaction terms were added to the regression models. An exploratory bootstrap indirect-association analysis with 5,000 resamples was conducted to assess whether the association between basic knowledge and basic skills was statistically consistent with an indirect association through basic concepts. This analysis used continuous domain scores and was estimated using a product-of-coefficients approach with bias-corrected and accelerated (BCa) bootstrap confidence intervals (25, 26). It was intended to describe statistical interrelations among simultaneously measured EHL domains rather than temporal or causal mediation. Because participants were recruited from two selected community sites, community site was included as a fixed-effect covariate in sensitivity analyses to assess whether the main findings were robust to between-community differences. Statistical significance was defined as two-sided *p* < 0.05.

## Results

### Participant characteristics

Among the 1,773 participants included in the final analysis, 953 (53.75%) were female. Most participants were aged 45 years or older (70.11%), married (83.93%), and had attained a college diploma or above (69.94%). Occupational exposure was reported by 120 participants (6.77%), and 89 participants (5.02%) reported access to occupational safety and health protection. All descriptive proportions, including the proportion of sufficient EHL, refer to the analytic community sample.

In crude comparisons, participants aged 18–44 years had higher mean EHL scores than those aged 45 years or older (84.76 ± 9.42 vs. 82.73 ± 11.45, *p* < 0.001). EHL also increased across categories of educational attainment (*p* < 0.001), rising from 75.73 ± 18.14 among participants with primary school education or below to 86.78 ± 8.44 among those with a master’s degree or above. Participants reporting occupational exposure had a higher crude mean score than those without reported exposure (85.41 ± 9.49 vs. 83.19 ± 11.00, *p* = 0.016). Mean EHL scores did not differ significantly by sex, marital status, or occupational safety and health protection (all *p* > 0.05; Table 1). When the national 70-point criterion was applied, 1,574 participants (88.8%) in the analytic sample had sufficient EHL. Because the study was conducted in two selected community survey sites without population weighting, this proportion was not interpreted as a city-level prevalence estimate. Significant differences in sufficient EHL were observed for age and education, whereas no significant differences were found for sex, marital status, occupational exposure, or occupational safety and health protection (Supplementary Table S1). Participants differed substantially between the two community sites (Supplementary Table S2). Compared with Fenglingnan Community, Xiulingbei Community had a higher mean total EHL score and a higher proportion of sufficient EHL. Significant between-community differences were also observed in marital status, educational attainment, occupational exposure, and occupational safety and health protection, whereas sex and age distribution did not differ significantly between the two community sites.

**Table 1:** Comparison of EHL scores across demographic and occupational characteristics (*n* = 1,773).

Variable	Category	*N* (%)	EHL Score (Mean ± SD)	*p*-value
Sex	Male	820 (46.25)	83.46 ± 10.52	0.662
	Female	953 (53.75)	83.23 ± 11.26	—
Age	18–44	530 (29.89)	84.76 ± 9.42	<0.001
	≥45 years	1,243 (70.11)	82.73 ± 11.45	—
Marital status	Married	1,488 (83.93)	83.21 ± 10.99	0.247
	Unmarried/divorced/widowed	285 (16.07)	84.00 ± 10.55	—
Education level	Primary school	49 (2.76)	75.73 ± 18.14	<0.001
	Junior high school	168 (9.48)	77.01 ± 14.83	—
	Senior high/vocational school	316 (17.82)	81.79 ± 11.33	—
	College diploma	293 (16.53)	82.46 ± 10.25	—
	Bachelor’s degree	795 (44.84)	85.42 ± 8.77	—
	Master’s degree or above	152 (8.57)	86.78 ± 8.44	—
Occupational exposure	Yes	120 (6.77)	85.41 ± 9.49	0.016
	No	1,653 (93.23)	83.19 ± 11.00	—
Occupational safety and health protection	Yes	89 (5.02)	84.27 ± 9.70	0.357
	No	1,684 (94.98)	83.29 ± 10.98	—

### Measurement properties of the EHL questionnaire

Internal consistency analysis showed a Cronbach’s alpha of 0.718 for the total 30-item EHL instrument. Domain-specific alpha coefficients were 0.389 for basic concepts, 0.534 for basic knowledge, and 0.592 for basic skills (Supplementary Table S3). Given the broad content coverage of the questionnaire, these coefficients were interpreted as descriptive measurement indicators rather than as formal psychometric validation.

### Education as the predominant independent predictor of EHL

To identify independent correlates of total EHL score, we fitted a multivariable linear regression model adjusting for age, sex, marital status, educational attainment, occupational exposure, and occupational safety and health protection. As shown in Table 2 (Model 1), educational attainment exhibited the clearest and most graded association with total EHL. Compared with participants with primary school education or below, those with senior high/vocational school education had a 6.28-point higher mean score (95% CI: 3.09 to 9.46; *p* < 0.001). The estimated difference increased across higher educational strata: 7.01 points for college diploma holders (95% CI: 3.77 to 10.24; *p* < 0.001), 9.99 points for bachelor’s degree holders (95% CI: 6.86 to 13.11; *p* < 0.001), and 11.32 points for participants with a master’s degree or above (95% CI: 7.84 to 14.80; *p* < 0.001).

**Table 2:** Multivariable linear regression models of EHL total score.

EHL	Variable	Category	*β* (95% CI)/Test statistic	*p*-value
Model 1	(Intercept)	—	74.76 (71.37, 78.14)	<0.001
	Age	≥45 years (ref: 18–44)	0.34 (−0.89, 1.57)	0.583
	Sex	Female (ref: Male)	0.51 (−0.49, 1.51)	0.315
	Marital status	Unmarried/divorced/widowed (ref: Married)	0.74 (−0.69, 2.17)	0.308
	Education level (ref: Primary school)	Junior high school	1.46 (−1.90, 4.82)	0.395
		Senior high/vocational school	6.28 (3.09, 9.46)	<0.001
		College diploma	7.01 (3.77, 10.24)	<0.001
		Bachelor’s degree	9.99 (6.87, 13.11)	<0.001
		Master’s degree or above	11.32 (7.84, 14.80)	<0.001
	Occupational Exposure	Yes (ref: No)	4.39 (0.64, 8.14)	0.022
	Occupational safety and health protection	Yes (ref: No)	−4.31 (−8.62, −0.01)	0.050
Model 2	(Intercept)	—	88.53 (81.92, 95.14)	<0.001
	Age	≥45 years (ref: 18–44)	0.33 (−0.90, 1.56)	0.599
	Marital status	Unmarried/divorced/widowed (ref: Married)	0.71 (−0.72, 2.14)	0.330
	Occupational Exposure	Yes (ref: No)	4.24 (0.50, 7.97)	0.026
	Occupational safety and health protection	Yes (ref: No)	−4.18 (−8.47, 0.10)	0.056
	Sex	Female (ref: Male)	−16.76 (−24.02, −9.50)	<0.001
	Education level (ref: Primary school)	Junior high school	−13.42 (−20.37, −6.47)	<0.001
		Senior high/vocational school	−7.65 (−14.40, −0.90)	0.026
		College diploma	−5.91 (−12.66, 0.84)	0.085
		Bachelor’s degree	−4.27 (−10.84, 2.30)	0.203
		Master’s degree or above	−1.82 (−8.67, 5.02)	0.602
	Sex × Education interaction (ref: Male × Primary)	Female × Junior high school	19.15 (11.21, 27.09)	<0.001
		Female × Senior high/vocational school	17.56 (9.93, 25.18)	<0.001
		Female × College diploma	15.76 (8.10, 23.43)	<0.001
		Female × Bachelor’s degree	18.30 (10.90, 25.70)	<0.001
		Female × Master’s degree or above	15.85 (7.83, 23.87)	<0.001
	Overall sex × education interaction	Model comparison test	*F* = 5.45, df = 5	<0.001

β = adjusted mean difference in health literacy total score; ref = reference category. Model 1 adjusted for age, sex, marital status, education level, occupational exposure, and occupational safety and health protection. Model 2 includes an interaction term between sex and education level. The overall interaction test was based on model comparison between models with and without sex-by-education interaction terms.

In contrast to the unadjusted analyses presented in Table 1, the age difference observed in crude analyses was fully attenuated after adjustment (*β* = 0.33; 95% CI: −0.90 to 1.56; *p* = 0.599). Sex, marital status, and occupational safety and health protection were not significantly associated with total EHL in the adjusted model (all *p* > 0.05). Occupational exposure remained statistically associated with total EHL (*β* = 4.24; 95% CI: 0.50 to 7.97; *p* = 0.026).

The logistic regression analysis using sufficient EHL as the outcome yielded a similar education gradient. Compared with participants with primary school education or below, those with senior high/vocational education had higher odds of sufficient EHL (aOR = 3.16; 95% CI: 1.56 to 6.23; *p* = 0.001). The adjusted odds ratios increased to 3.43 for college diploma holders (95% CI: 1.65 to 6.96; *p* < 0.001), 8.16 for bachelor’s degree holders (95% CI: 3.95 to 16.52; *p* < 0.001), and 10.06 for participants with a master’s degree or above (95% CI: 3.82 to 28.98; *p* < 0.001). Junior high school education was not significantly associated with sufficient EHL (aOR = 1.14; 95% CI: 0.56 to 2.25; *p* = 0.712). Age, sex, marital status, occupational exposure, and occupational safety and health protection were not significant in this model (Supplementary Table S4). In sensitivity analyses additionally adjusting for community site, the educational gradient remained evident for both continuous total EHL score and sufficient EHL (Supplementary Table S4). For total EHL score, the adjusted mean differences remained significant for senior high/vocational education, college diploma, bachelor’s degree, and master’s degree or above. Similarly, the odds of sufficient EHL remained higher among participants with senior high/vocational education or above after adjustment for community site. The positive association between occupational exposure and continuous total EHL score was attenuated after community adjustment, whereas the main educational pattern remained stable.

### EHL subdomain scores and sex comparison

In the total sample, the mean domain scores were 16.46 ± 2.49 for basic concepts, 42.33 ± 6.59 for basic knowledge, and 24.54 ± 4.12 for basic skills. No statistically significant sex differences were observed in any domain: basic concepts (16.57 ± 2.43 in males vs. 16.37 ± 2.55 in females; *p* = 0.091), basic knowledge (42.39 ± 6.35 vs. 42.27 ± 6.79; *p* = 0.688), or basic skills (24.49 ± 4.02 vs. 24.59 ± 4.20; *p* = 0.613) (Table 3).

**Table 3:** Comparison of basic concepts, basic knowledge, and basic skills scores by sex.

Variable	Total (Mean ± SD)	Male (Mean ± SD)	Female (Mean ± SD)	*p*-value
Basic concepts	16.46 ± 2.49	16.57 ± 2.43	16.37 ± 2.55	0.091
Basic knowledge	42.33 ± 6.59	42.39 ± 6.35	42.27 ± 6.79	0.688
Basic skills	24.54 ± 4.12	24.49 ± 4.02	24.59 ± 4.20	0.613

Multivariable domain-specific models further showed that education was most strongly associated with basic knowledge and basic skills (Table 4). In Model 1, participants with a master’s degree or above had the largest increase in basic knowledge score compared with the primary school reference group (*β* = 7.13; 95% CI: 5.03 to 9.24; *p* < 0.001). A similar, although smaller, gradient was observed for basic skills, with the highest education group scoring 3.06 points higher than the reference group (95% CI: 1.74 to 4.38; *p* < 0.001). Occupational exposure was positively associated with basic knowledge in the adjusted model (*β* = 2.81; 95% CI: 0.55 to 5.08; *p* = 0.015), whereas occupational safety and health protection was inversely associated with basic knowledge (*β* = −2.72; 95% CI: −5.32 to −0.13; *p* = 0.040).

**Table 4:** Associations of sociodemographic and occupational factors with basic concepts, basic knowledge, and basic skills.

Variable	Category	Basic Concepts		Basic Knowledge		Basic Skills	
		Model 1	Model 2	Model 1	Model 2	Model 1	Model 2
Intercept	—	15.47 (14.67, 16.27) < 0.001	16.48 (14.91, 18.05) < 0.001	37.22 (35.18, 39.26) < 0.001	45.35 (41.35, 49.34) < 0.001	22.06 (20.77, 23.35) < 0.001	26.70 (24.17, 29.24) < 0.001
Age	≥45 years	0.25 (−0.04, 0.54) 0.091	0.25 (−0.04, 0.54) 0.090	−0.27 (−1.01, 0.47) 0.478	−0.29 (−1.03, 0.45) 0.449	0.36 (−0.12, 0.84) 0.131	0.36 (−0.11, 0.83) 0.129
Sex	Female	−0.13 (−0.37, 0.11) 0.289	−1.39 (−3.11, 0.34) 0.114	0.31 (−0.29, 0.91) 0.317	−9.87 (−14.25, −5.49) < 0.001	0.33 (−0.05, 0.71) 0.089	−5.51 (−8.28, −2.73) < 0.001
Marital status	Unmarried/divorced/widowed	0.37 (0.03, 0.71) 0.030	0.36 (0.02, 0.70) 0.039	0.22 (−0.64, 1.08) 0.625	0.19 (−0.67, 1.06) 0.658	0.16 (−0.40, 0.72) 0.578	0.16 (−0.39, 0.70) 0.570
Education (ref: Primary)	Junior high	0.21 (−0.58, 1.00) 0.608	−0.76 (−2.41, 0.88) 0.363	1.48 (−0.55, 3.50) 0.154	−7.41 (−11.60, −3.22) < 0.001	−0.22 (−1.51, 1.07) 0.733	−5.25 (−7.91, −2.58) < 0.001
	Senior high/vocational	0.66 (−0.09, 1.41) 0.084	−0.33 (−1.93, 1.27) 0.685	4.07 (2.15, 5.98) < 0.001	−4.23 (−8.30, −0.16) 0.042	1.55 (0.33, 2.76) 0.013	−3.09 (−5.67, −0.51) 0.019
	College diploma	0.47 (−0.29, 1.23) 0.229	−0.18 (−1.77, 1.42) 0.828	4.73 (2.78, 6.68) < 0.001	−2.94 (−7.01, 1.14) 0.157	1.81 (0.57, 3.06) 0.004	−2.80 (−5.38, −0.22) 0.033
	Bachelor’s	1.07 (0.34, 1.80) 0.004	−0.07 (−1.63, 1.48) 0.925	6.20 (4.32, 8.08) < 0.001	−2.15 (−6.12, 1.81) 0.288	2.72 (1.52, 3.90) < 0.001	−2.04 (−4.56, 0.48) 0.112
	Master’s or above	1.13 (0.31, 1.94) 0.007	0.08 (−1.54, 1.70) 0.925	7.13 (5.03, 9.24) < 0.001	−0.55 (−4.69, 3.59) 0.794	3.06 (1.74, 4.38) < 0.001	−1.35 (−3.98, 1.28) 0.314
Occupational exposure	Yes	0.71 (−0.17, 1.60) 0.113	0.71 (−0.18, 1.59) 0.117	2.81 (0.55, 5.08) 0.015	2.71 (0.46, 4.97) 0.018	0.86 (−0.58, 2.30) 0.238	0.81 (−0.62, 2.25) 0.264
Occupational safety and health protection	Yes	−0.32 (−1.34, 0.69) 0.531	−0.33 (−1.34, 0.68) 0.523	−2.72 (−5.32, −0.13) 0.040	−2.63 (−5.22, −0.05) 0.046	−1.27 (−2.91, 0.37) 0.132	−1.22 (−2.86, 0.42) 0.145
Sex × Education	Female × Junior high	—	1.20 (−0.68, 3.09) 0.210	—	11.48 (6.68, 16.28) < 0.001	—	6.47 (3.42, 9.51) < 0.001
	Female × Senior high	—	1.24 (−0.57, 3.05) 0.179	—	10.49 (5.88, 15.10) < 0.001	—	5.82 (2.90, 8.75) < 0.001
	Female × College	—	0.61 (−1.20, 2.42) 0.511	—	9.38 (4.76, 14.00) < 0.001	—	5.78 (2.85, 8.71) < 0.001
	Female × Bachelor’s	—	1.55 (−0.20, 3.30) 0.084	—	10.68 (6.21, 15.15) < 0.001	—	6.07 (3.23, 8.91) < 0.001
	Female × Master’s+	—	1.36 (−0.54, 3.27) 0.158	—	9.19 (4.36, 14.02) < 0.001	—	5.30 (2.23, 8.37) < 0.001

Each cell shows β (95% CI) on the first line and p-value on the second line. Significant associations (p < 0.05) are in bold. “—” indicates the term was not included in Model 1. All models adjusted for age, marital status, occupational exposure, and occupational protection. In Model 2, main effects of sex and education are conditional on the reference group (Male × Primary school). Overall sex-by-education interaction tests were conducted by comparing models with and without sex-by-education interaction terms. The overall interaction was not statistically significant for basic concepts (*F* = 1.98, df = 5, *p* = 0.079), but was significant for basic knowledge (*F* = 5.19, df = 5, *p* < 0.001) and basic skills (*F* = 3.86, df = 5, *p* = 0.002).

### Sex-specific associations by education level

A statistically significant overall sex-by-education interaction was observed for total EHL score (overall interaction test: *F* = 5.45, df = 5, *p* < 0.001; Table 2). Within the reference stratum of primary school education or below, the estimated main effect for female participants was negative (*β* = −16.76; 95% CI: −24.02 to −9.50). In contrast, the interaction terms between female sex and higher educational levels were positive and statistically significant. The largest early shift was observed among female participants with junior high school education (*β* for interaction = 19.15; 95% CI: 11.21 to 27.09).

Domain-specific analyses further showed significant overall sex-by-education interactions for basic knowledge (*F* = 5.19, df = 5, *p* < 0.001) and basic skills (*F* = 3.86, df = 5, *p* = 0.002), but not for basic concepts (*F* = 1.98, df = 5, *p* = 0.079; Table 4). For basic knowledge, the interaction coefficient was 11.48 (95% CI: 6.68 to 16.28) for female participants with junior high school education and 9.19 (95% CI: 4.36 to 14.02) for those with a master’s degree or above. A parallel pattern was observed for basic skills, with interaction coefficients of 6.47 (95% CI: 3.42 to 9.51) and 5.30 (95% CI: 2.23 to 8.37), respectively. The sex-by-education interaction was also robust in the sensitivity analysis additionally adjusting for community site. The overall interaction remained statistically significant for total EHL score, and the female-by-education interaction coefficients remained positive across all higher educational categories (Supplementary Table S5).

### Exploratory indirect-association analysis by sex

Exploratory indirect-association analysis was used to examine whether the association between basic knowledge and basic skills was statistically consistent with an indirect association through basic concepts (Tables 5, 6 and Figure 1). In the overall sample, the indirect association was small but positive (indirect association = 0.005; 95% BCa bootstrap CI: 0.001 to 0.012). In sex-stratified analyses, the indirect association was observed among women (indirect association = 0.009; 95% BCa bootstrap CI: 0.004 to 0.016), whereas the confidence interval for men included zero, suggesting limited evidence of an indirect-association pattern in this subgroup. The fitted regression lines further showed positive associations across EHL subdomains in both sexes, with a stronger knowledge-to-skills association among women (Figure 2).

**Table 5:** Exploratory indirect-association analysis: Basic concepts as a statistical link between basic knowledge and basic skills.

Path / Effect	Estimate	SE	95% CI
Direct paths			
Basic knowledge → Basic concepts (a)	0.010	0.005	[0.001, 0.019]
Basic concepts → Basic skills (b)	0.488	0.124	[0.254, 0.739]
Basic knowledge → Basic skills (c)	0.230	0.016	[0.198, 0.261]
Indirect associations			
Indirect association (a × b)	0.005	0.003	[0.001, 0.012]
Total association (c + a × b)	0.235	0.016	[0.204, 0.266]

All estimates are unstandardized coefficients from the indirect-association analysis fitted with bootstrap (5,000 replications). The indirect association was assessed using the bias-corrected accelerated (BCa) bootstrap 95% confidence interval.

**Table 6:** Sex-specific indirect associations of basic knowledge with basic skills through basic concepts.

Path / Effect	Male (n = 820)	Female (n = 953)
a: Basic knowledge → Basic concepts	0.006 [−0.007, 0.018]	0.016 [0.002, 0.029]
b: Basic concepts → Basic skills	0.402 [0.108, 0.747]	0.588 [0.225, 0.955]
c: Conditional direct association	0.234 [0.190, 0.275]	0.231 [0.187, 0.277]
Indirect association (a × b)	0.002 [0.000, 0.006]	0.009 [0.004, 0.016]

All coefficients are unstandardized. Confidence intervals are bias-corrected and accelerated (BCa) 95% CIs based on 5,000 bootstrap samples. The indirect association was considered statistically evident if the 95% CI does not include zero.

**Figure 1:**
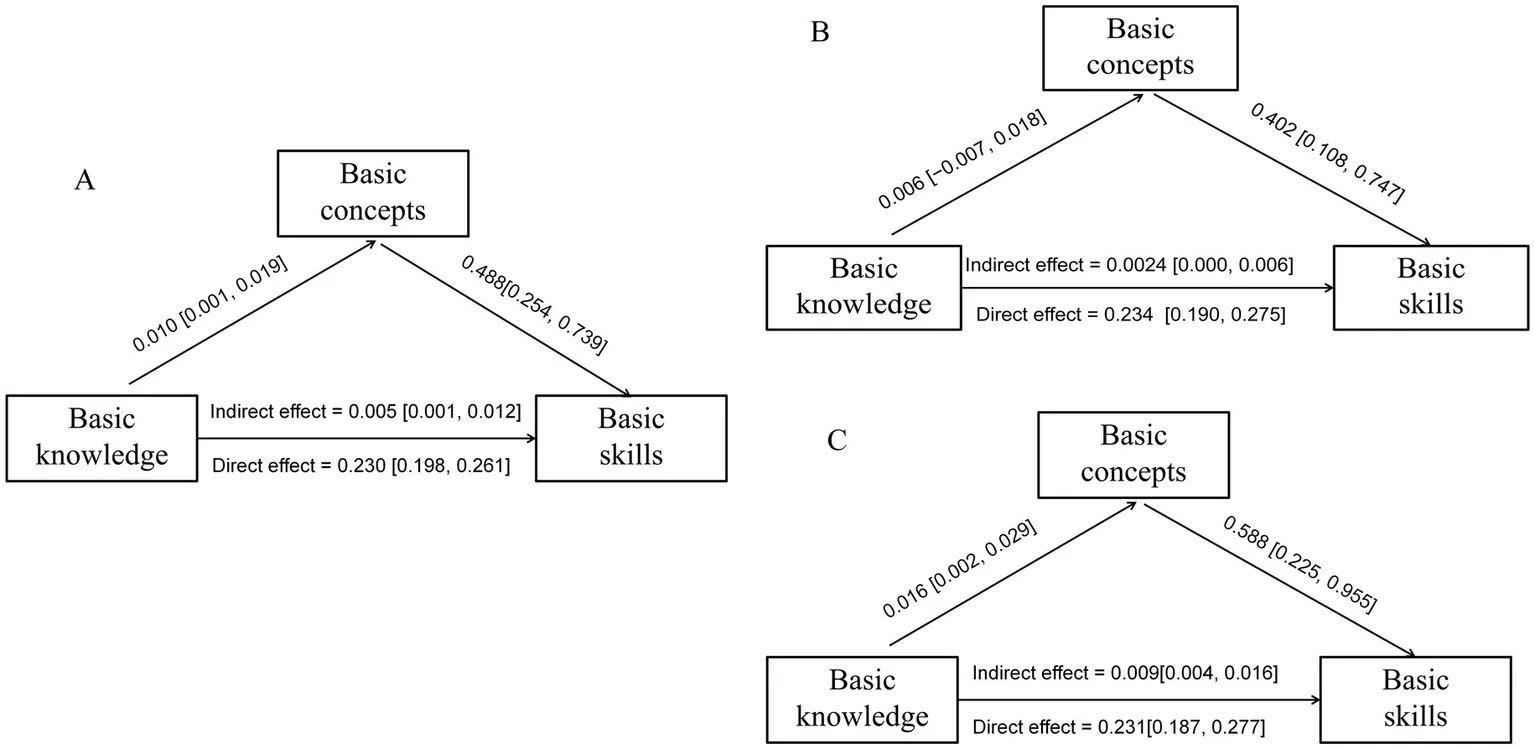
Exploratory indirect association between basic knowledge and basic skills through basic concepts in the total sample **(A)**, male participants **(B)**, and female participants **(C)**, Values are unstandardized coefficients with bias-corrected and accelerated bootstrap 95% confidence intervals. Arrows represent prespecified statistical associations and should not be interpreted as temporal or causal pathways.

**Figure 2:**
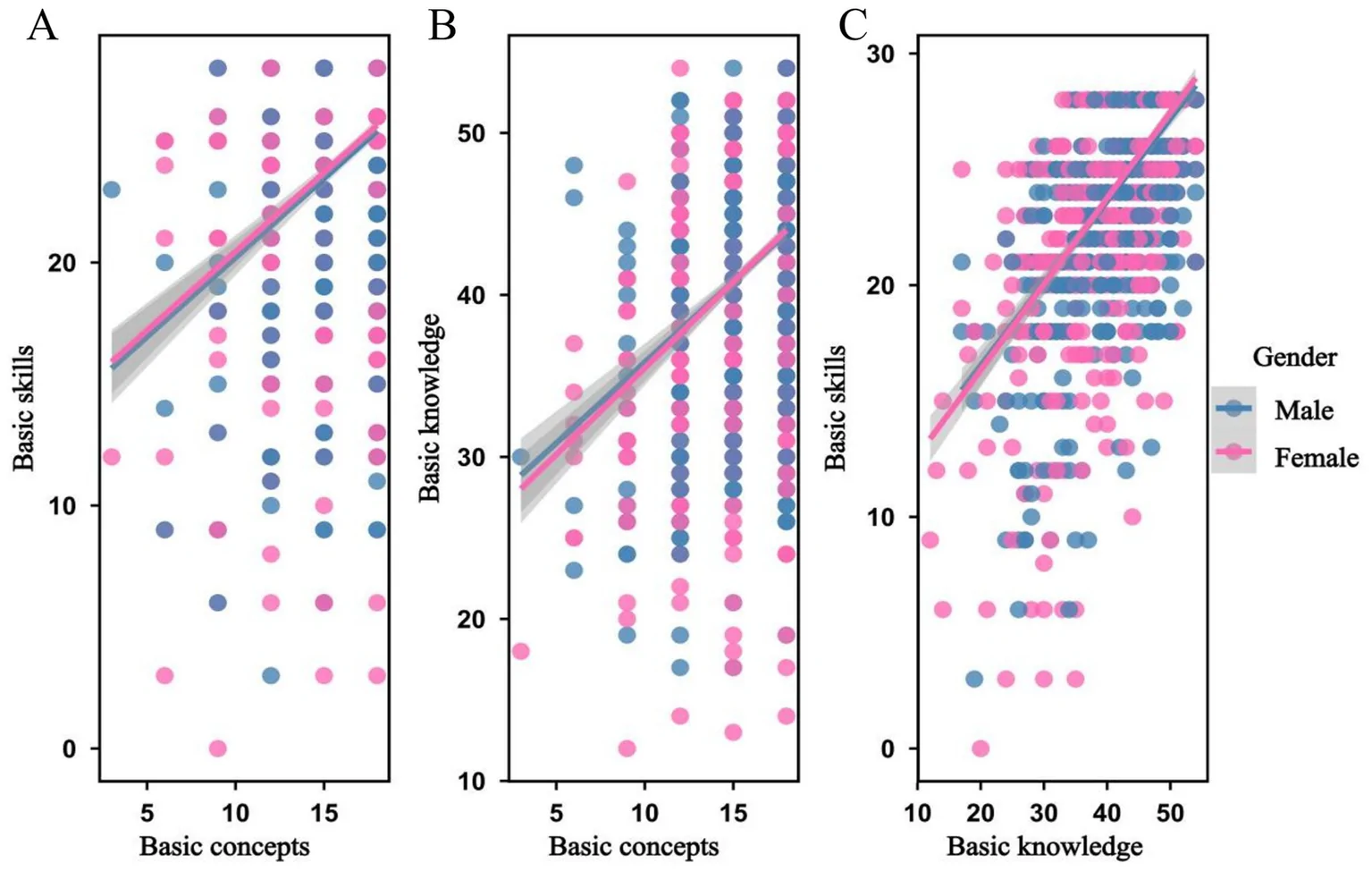
Sex-specific cross-sectional associations among environmental health literacy domains. Panel A shows the association between basic concepts and basic skills; Panel B shows the association between basic concepts and basic knowledge; and Panel C shows the association between basic knowledge and basic skills. Points represent individual participants. Colored lines represent sex-specific fitted linear regression lines, and shaded areas indicate 95% confidence intervals. Blue represents male participants and pink represents female participants.

## Discussion

This community-based survey of 1,773 adults recruited from two selected urban communities in Nanning provides evidence that EHL is associated with educational resources and statistically linked EHL domains within the analytic sample. Because the study was not based on probability sampling and no population weights were available, the observed EHL distribution and the 88.8% sufficient-EHL proportion should not be interpreted as population-representative estimates for all adults in Nanning. Instead, the main contribution of this study lies in characterizing education-related differences, sex-by-education heterogeneity, and cross-sectional interrelations among predefined EHL domains in selected urban community settings.

The strong educational gradient observed in this study is consistent with broader health-literacy and EHL evidence showing that formal education strengthens information acquisition, reading comprehension, scientific reasoning, and risk appraisal (27, 28). In the context of environmental health, these competencies are particularly important because residents are often required to interpret fragmented and sometimes technical information about air pollution, water safety, chemicals, radiation, climate change, and occupational hazards. The attenuation of the age association after educational adjustment further suggests that the apparent disadvantage among older adults may partly reflect cohort differences in educational access rather than ageing per se. This interpretation is important for intervention design: age-targeted communication alone may be insufficient unless it also addresses the more fundamental educational and informational barriers that constrain environmental health understanding.

A notable finding was the significant interaction between sex and educational attainment. In the lowest educational stratum, women had lower estimated EHL scores than men, whereas the education-related increase in EHL was steeper among women, particularly for basic knowledge and basic skills. This result should not be interpreted as an intrinsic sex difference in learning ability. Instead, it is more plausibly understood through an intersectional lens: sex and education may jointly define access to information, confidence in interpreting scientific content, and opportunities to apply environmental health knowledge in daily life (29, 30). In settings where women assume substantial responsibility for household health management, caregiving, food safety, cleaning practices, and the protection of children or older family members, environmental health information may become more behaviorally salient once formal education has provided the cognitive tools needed to interpret it (31–34). Conversely, women with very limited schooling may experience compounded disadvantages, including restricted access to formal knowledge systems, digital resources, and social channels for risk communication. Because the primary-school reference group was small, this interaction should be interpreted as evidence of heterogeneity in education-related EHL patterns rather than as a definitive statement about sex differences within a single educational category.

The exploratory indirect-association analysis provides a cautious domain-level interpretation of this social pattern. Basic concepts were statistically consistent with an indirect link between basic knowledge and basic skills, especially among women. This finding suggests that factual knowledge may be more closely connected to practical environmental health capacity when organized through foundational concepts, such as the relationship between environmental exposures and health outcomes, the preventability of risk, and individual and collective responsibility for environmental protection (20, 35). Because the data are cross-sectional and all EHL domains were measured simultaneously, this finding should not be interpreted as causal mediation. Rather, it generates a hypothesis that should be tested using longitudinal designs or intervention studies.

The occupational findings require more cautious interpretation than the education- and sex-related results. Occupational exposure was positively associated with total EHL and basic knowledge in the primary adjusted models, but the association with total EHL was attenuated after community site was additionally considered in the sensitivity analysis. This suggests that the observed occupational association may partly reflect differences in participant composition or community context rather than a stable independent effect of occupational exposure. Thus, the finding should not be interpreted as evidence that exposure improves EHL. The present survey did not collect detailed information on occupation type, exposure intensity, duration of exposure, safety training, or the specific content of protective measures. Therefore, the mechanisms underlying these occupational findings cannot be clarified.

Taken together, these findings have several implications for environmental health promotion. First, interventions should prioritize residents with limited formal education, because educational disparities appear to structure both overall EHL and the knowledge-to-skill association. Communication materials for these groups should avoid excessive technicality and should translate key concepts into concrete scenarios, such as indoor ventilation, drinking-water safety, waste sorting, heat exposure, and chemical-use precautions. Second, concept-centered education may be more effective than fact-based messaging alone. Strengthening residents’ understanding of exposure–health relationships may improve their ability to generalize knowledge across different hazards. Third, sex-responsive strategies should be designed without reinforcing stereotypes: for women, household and caregiving contexts may provide useful entry points for skill-oriented interventions; for men, occupational, community, or task-based settings may be more effective channels for transforming risk knowledge into protective practices. Finally, workplaces should combine procedural safety requirements with participatory and explanatory training so that protection is not reduced to compliance, but becomes part of workers’ broader environmental health competence.

### Limitations

Several limitations should be acknowledged. First, this study was conducted in two selected urban communities and did not use a province-wide or city-wide complex probability sampling design. No household sampling frame was established, households were not sampled as a separate stage, and sampling weights were not available. Therefore, the findings cannot be generalized to all adults in Nanning. Second, although completed and valid questionnaires were documented, field records did not include a complete contemporaneous tally of all residents approached and all refusals; therefore, a formal response rate could not be reconstructed. Because participation depended on residents being present at designated community survey sites and willing to complete a face-to-face questionnaire, volunteer bias and availability bias may have influenced the observed EHL level and its correlates. Third, the relatively high proportion of participants with college-level education or above may have contributed to the high observed proportion of sufficient EHL; this proportion should therefore be interpreted as sample-specific rather than population-representative. Fourth, the primary-school reference group was small (*n* = 49), which may have reduced the precision of regression estimates and increased the instability of sex-by-education interaction estimates. Fifth, although the total instrument showed acceptable internal consistency, the domain-specific Cronbach’s alpha coefficients were modest, particularly for basic concepts. Because the questionnaire was designed as a content-based assessment covering heterogeneous EHL topics, domain-level coefficients were interpreted descriptively rather than as definitive evidence of construct validity. Finally, the cross-sectional design precludes causal inference, and the observed associations, interactions, and indirect-association patterns should not be interpreted as temporal or causal pathways.

## Conclusion

Educational attainment was the strongest correlate of EHL in this selected urban community sample. The observed sex-by-education interaction and exploratory indirect-association patterns suggest that basic concepts may be statistically linked to the association between environmental health knowledge and practical skills, particularly among women. These findings support education-sensitive, concept-centered, and skill-oriented EHL promotion strategies, while emphasizing the need for probability-based and longitudinal studies to assess generalizability and temporal ordering.

### Public health implications

For future research, probability-based sampling, standardized response-rate recording, and longitudinal designs are needed to evaluate the generalizability and temporal ordering of EHL domains. For policymakers, EHL promotion should prioritize populations with limited formal education and should avoid interpreting community-sample proportions as city-level prevalence estimates. For public health practitioners, EHL assessment can be integrated into community health education and environmental risk communication. For community educators, interventions should emphasize practical skills, including information appraisal, protective behaviors, and emergency responses, while using accessible, scenario-based communication materials.

## Data Availability

The de-identified data supporting the findings of this study are available from the corresponding author upon reasonable request, subject to applicable ethical, institutional, and data-protection requirements.
