# Supplementary material for: Educational gradients and sex-by-education differences in environmental health literacy among adults from selected urban communities in Nanning, China
Source: Front Public Health. 2026 Oct 2;14:1928358. doi: 10.3389/fpubh.2026.1928358 (PMC13672492; doi:10.3389/fpubh.2026.1928358)
Supplement: Supplementary file 1 [file Supplementary_file_1.docx]

| Table S1 Sample characteristics and sufficient EHL in the participants (n=1773) | | | | | |
| --- | --- | --- | --- | --- | --- |
| Variable | Category | N | Level of EHL | χ² | p-value |
| Sex | Male | 820 | 732 (89.27%) | 0.28 | 0.594 |
|  | Female | 953 | 842 (88.35%) |  |  |
| Age | 18-44 | 530 | 493 (93.02%) | 13.06 | <0.001 |
|  | ≥45 | 1243 | 1081 (86.97%) |  |  |
| Marital status | Married | 1488 | 1318 (88.58%) | 0.26 | 0.610 |
|  | Unmarried/divorced/widowed | 285 | 256 (89.82%) |  |  |
| Education level | Primary school | 49 | 33 (67.35%) | 118.07 | <0.001 |
|  | Junior high school | 168 | 117 (69.64%) |  |  |
|  | Senior high/vocational school | 316 | 273 (86.39%) |  |  |
|  | College diploma | 293 | 256 (87.37%) |  |  |
|  | Bachelor’s degree | 795 | 750 (94.34%) |  |  |
|  | Master’s degree or above | 152 | 145 (95.39%) |  |  |
| Occupational exposure | Yes | 120 | 110 (91.67%) | 0.79 | 0.374 |
|  | No | 1653 | 1464 (88.56%) |  |  |
| Occupational safety and health protection | Yes | 89 | 80 (89.89%) | 0.03 | 0.866 |
|  | No | 1684 | 1494 (88.72%) |  |  |

| Table S2. Participant characteristics and environmental health literacy by community site | | | | |
| --- | --- | --- | --- | --- |
| Variable | Category | Fenglingnan Community  (n = 828) | Xiulingbei Community  (n = 945) | P value |
| Sex | Male | 373 (45.05) | 447 (47.30) | 0.34 |
|  | Female | 455 (54.95) | 498 (52.70) |  |
| Age group | 18–44 years | 243 (29.35) | 287 (30.37) | 0.64 |
|  | ≥45 years | 585 (70.65) | 658 (69.63) |  |
| Marital status | Married | 741 (89.49) | 747 (79.05) | <0.001 |
|  | Unmarried/divorced/widowed | 87 (10.51) | 198 (20.95) |  |
| Education level | Primary school | 35 (4.23) | 14 (1.48) | <0.001 |
|  | Junior high school | 111 (13.41) | 57 (6.03) |  |
|  | Senior high/vocational school | 150 (18.12) | 166 (17.57) |  |
|  | College diploma | 150 (18.12) | 143 (15.13) |  |
|  | Bachelor’s degree | 342 (41.30) | 453 (47.94) |  |
|  | Master’s degree or above | 40 (4.83) | 112 (11.85) |  |
| Occupational exposure | No | 809 (97.71) | 844 (89.31) | <0.001 |
|  | Yes | 19 (2.29) | 101 (10.69) |  |
| Occupational safety and health protection | No | 815 (98.43) | 869 (91.96) | <0.001 |
|  | Yes | 13 (1.57) | 76 (8.04) |  |
| Sufficient EHL | No | 156 (18.84) | 43 (4.55) | <0.001 |
|  | Yes | 672 (81.16) | 902 (95.45) |  |
| Total EHL score | Mean ± SD | 80.69 ± 12.90 | 85.66 ± 8.16 | <0.001 |
| Note: Values are presented as n (%) unless otherwise indicated. EHL, environmental health literacy. P values were derived from chi-square tests for categorical variables and independent-samples t tests for continuous variables. | | | | |

Table S3. Internal consistency of the EHL questionnaire

| Scale/domain | Number of items | Cronbach’s alpha |
| --- | --- | --- |
| Total EHL instrument | 30 | 0.718 |
| Basic concepts | 6 | 0.389 |
| Basic knowledge | 15 | 0.534 |
| Basic skills | 9 | 0.592 |

| Table S4. Sensitivity analysis additionally adjusting for community site | | |
| --- | --- | --- |
| Outcome and variable | Main model | Community-adjusted model |
| Total EHL score, β (95% CI) |  |  |
| Age group: ≥45 years, ref: 18–44 years | 0.34 (-0.89 to 1.57) | -0.08 (-1.29 to 1.13) |
| Female, ref: male | 0.51 (-0.49 to 1.51) | 0.53 (-0.46 to 1.51) |
| Unmarried/divorced/widowed, ref: married | 0.74 (-0.69 to 2.17) | -0.20 (-1.63 to 1.23) |
| Junior high school, ref: primary school | 1.46 (-1.90 to 4.82) | 1.11 (-2.20 to 4.42) |
| Senior high/vocational school, ref: primary school | 6.28 (3.09 to 9.46) | 5.14 (2.00 to 8.29) |
| College diploma, ref: primary school | 7.01 (3.77 to 10.24) | 5.97 (2.78 to 9.16) |
| Bachelor’s degree, ref: primary school | 9.99 (6.86 to 13.11) | 8.63 (5.54 to 11.72) |
| Master’s degree or above, ref: primary school | 11.32 (7.84 to 14.80) | 9.30 (5.84 to 12.76) |
| Occupational exposure: yes, ref: no | 4.39 (0.64 to 8.14) | 3.26 (-0.44 to 6.96) |
| Occupational safety and health protection: yes, ref: no | -4.31 (-8.62 to -0.01) | -4.40 (-8.64 to -0.17) |
| Sufficient EHL, OR (95% CI) |  |  |
| Age group: ≥45 years, ref: 18–44 years | 0.94 (0.60 to 1.45) | 0.85 (0.54 to 1.32) |
| Female, ref: male | 1.10 (0.80 to 1.51) | 1.13 (0.82 to 1.56) |
| Unmarried/divorced/widowed, ref: married | 1.08 (0.69 to 1.74) | 0.81 (0.51 to 1.33) |
| Junior high school, ref: primary school | 1.14 (0.56 to 2.25) | 1.04 (0.50 to 2.11) |
| Senior high/vocational school, ref: primary school | 3.16 (1.56 to 6.23) | 2.37 (1.14 to 4.81) |
| College diploma, ref: primary school | 3.43 (1.65 to 6.96) | 2.69 (1.27 to 5.60) |
| Bachelor’s degree, ref: primary school | 8.16 (3.95 to 16.52) | 5.98 (2.82 to 12.44) |
| Master’s degree or above, ref: primary school | 10.06 (3.82 to 28.98) | 5.68 (2.09 to 16.81) |
| Occupational exposure: yes, ref: no | 3.00 (0.61 to 54.36) | 1.91 (0.38 to 34.73) |
| Occupational safety and health protection: yes, ref: no | 0.29 (0.02 to 1.72) | 0.27 (0.01 to 1.65) |
| Note: EHL, environmental health literacy; CI, confidence interval; OR, odds ratio. The main models adjusted for age group, sex, marital status, educational attainment, occupational exposure, and occupational safety and health protection. The community-adjusted models additionally included community site as a fixed-effect covariate. | | |

| Table S5. Sensitivity analysis of the sex-by-education interaction after adjustment for community site | | | |
| --- | --- | --- | --- |
| Interaction term | β | 95% CI | P value |
| Female × Junior high school | 18.82 | 11.01 to 26.64 | <0.001 |
| Female × Senior high/vocational school | 16.67 | 9.15 to 24.18 | <0.001 |
| Female × College diploma | 15.31 | 7.78 to 22.84 | <0.001 |
| Female × Bachelor’s degree | 17.44 | 10.15 to 24.73 | <0.001 |
| Female × Master’s degree or above | 15.52 | 7.65 to 23.40 | <0.001 |
| Overall sex × education interaction | F = 5.11 | df = 5 | <0.001 |
| Note: The model adjusted for age group, marital status, occupational exposure, occupational safety and health protection, and community site. Primary school education among men was the reference category for the interaction terms. β represents the additional difference in total EHL score for women in each education category relative to the corresponding male-by-primary-school reference structure. The overall interaction test was based on model comparison between models with and without sex-by-education interaction terms. | | | |

Figure S1. Distribution of total EHL score in the analytic sample.
